# Multimodal deep learning from satellite and street-level imagery for measuring income, overcrowding, and environmental deprivation in urban areas

**DOI:** 10.1016/j.rse.2021.112339

**Published:** 2021-05

**Authors:** Esra Suel, Samir Bhatt, Michael Brauer, Seth Flaxman, Majid Ezzati

**Affiliations:** a MRC Centre for Environment and Health, School of Public Health, Imperial College London, London, UK; b Swiss Data Science Center, ETH Zurich and EPFL, Switzerland; c MRC Centre for Global Infectious Disease Analysis, School of Public Health, Imperial College London, London, UK; d Section of Epidemiology, Department of Public Health, University of Copenhagen, Denmark; e Abdul Latif Jameel Institute for Disease and Emergency Analytics, Imperial College London, London, UK; f School of Population and Public Health, University of British Columbia, Vancouver, British Columbia, Canada; g Institute for Health Metrics & Evaluation, University of Washington, Seattle, WA, USA; h Department of Mathematics, Imperial College London, London, UK; i Regional Institute for Population Studies, University of Ghana, Accra, Ghana

**Keywords:** Convolutional neural networks, Segmentation, Urban measurements, Satellite images, Street-level images

## Abstract

Data collected at large scale and low cost (e.g. satellite and street level imagery) have the potential to substantially improve resolution, spatial coverage, and temporal frequency of measurement of urban inequalities. Multiple types of data from different sources are often available for a given geographic area. Yet, most studies utilize a single type of input data when making measurements due to methodological difficulties in their joint use. We propose two deep learning-based methods for jointly utilizing satellite and street level imagery for measuring urban inequalities. We use London as a case study for three selected outputs, each measured in decile classes: income, overcrowding, and environmental deprivation. We compare the performances of our proposed multimodal models to corresponding unimodal ones using mean absolute error (MAE). First, satellite tiles are appended to street level imagery to enhance predictions at locations where street images are available leading to improvements in accuracy by 20, 10, and 9% in units of decile classes for income, overcrowding, and living environment. The second approach, novel to the best of our knowledge, uses a U-Net architecture to make predictions for all grid cells in a city at high spatial resolution (e.g. for 3 m × 3 m pixels in London in our experiments). It can utilize city wide availability of satellite images as well as more sparse information from street-level images where they are available leading to improvements in accuracy by 6, 10, and 11%. We also show examples of prediction maps from both approaches to visually highlight performance differences.

## Highlights

• Our model utilizes information from street-level and satellite images.
• Proposed multimodal measurement approaches outperform unimodal ones.
• The model can deal with missing data during training and predictions.
• Multimodal frameworks can incorporate additional modalities (e.g. aerial images).
• Applications can be expanded to different outcomes.

## Graphical abstract

**Figure:**
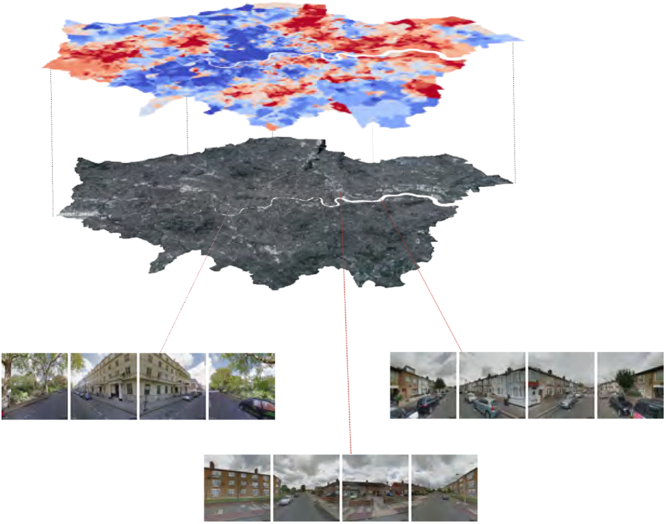

## 1 Introduction

Over half of the global population is currently urban, with urban areas projected to absorb all future population growth. As cities adapt to growth in population and increasing density, challenges and conflicts emerge regarding provision of services, such as adequate and affordable housing and access to health care, leading to increasing and dramatic levels of inequality. Reducing inequalities is integral to the global sustainable development agenda and to local city policies (Lu et al., 2015; GLA, 2017, GLA, 2018). However, data for informing these policies and measuring their actual impacts, are currently only available from disjointed, and inefficient surveillance systems and may not be available over sufficient time periods to best inform interventions. Measuring socioeconomic status (SES) and its different dimensions at high spatial and temporal resolution is crucial yet poses a significant challenge, for example relying on periodic census information collected at 5 or 10 year intervals. Emerging sources of large-scale data, such as remote sensing, street-level imagery, mobile phones, and crowd-sourced data, coupled with advances in deep learning methods, have the potential to significantly advance the speed, frequency and spatial precision of the measurement of urban characteristics. Such advances may help identify specific areas of concern at earlier stages, so that interventions can be more quickly implemented through targeting policies to areas of greatest need.

Researchers are increasingly interested in leveraging rapidly expanding availability of geospatial and remote sensing image data and advances in deep learning (Weichenthal et al., 2019). Relevant applications of machine learning on imagery include detecting from satellite data: poverty (Jean et al., 2016; Steele et al., 2017; Xie et al., 2015; Jean et al., 2018; You et al., 2017), air pollution (Hong et al., 2019), harvest size and crop yield (Lobell, 2013; You et al., 2017), and from street level images: income (Gebru et al., 2017), perceived safety (Naik et al., 2014, Naik et al., 2017), greenness and openness (Seiferling et al., 2017; Richards and Edwards, 2017; Larkin and Hystad, 2019), housing prices (Law et al., 2019; Glaeser et al., 2018), air pollution (Apte et al., 2017), social and environmental inequalities (Suel et al., 2019, Suel et al., 2018).

Different sources of data have different strengths and weaknesses. Street level and satellite images essentially contain visual information captured from different viewpoints about our environment. Street level images are captured by cameras mounted on cars, bikes, or backpacks; each image corresponds to a photo taken at geographically fixed locations and is rich in local information content. However, these images are usually taken on a specific time for selected locations from the vantage point of the street only and with irregular intervals in different seasons, times of day or days of the week. Images captured by satellites orbiting the Earth, on the other hand, have a specific spatial resolution which determines the surface area represented by each pixel (e.g. 3*m*^2^ for the data used in our experiments). Such images are typically available at periodic intervals (e.g. once per day or increasingly more frequently) and their coverage is much higher. Remote sensing data typically includes visual information as well as data from other portions of the electromagnetic spectrum. They capture a bird's eye view point which may have less information content compared to street-level images for some outcomes.

Existing studies are mostly focused on the use of a single source of imagery data for these measurement tasks hence cannot jointly use information from different data sources. Methods are needed to better utilize all available information from different modalities. In this study, we propose two approaches for jointly utilizing imagery data captured at different scales and view points, with varying spectral capabilities and resolutions: street-level images and satellite data. We demonstrate our methods on satellite and street level imagery from London where the aim is to predict income, overcrowding, and environmental deprivation at high spatial resolution and coverage. The same general methodology could be applied to other applications, as well as to multimodal settings where additional sources of images are available (e.g. aerial, satellite images at different resolutions). In our experiments, we also compare performance of our proposed bimodal method with unimodal learning methods that rely only on satellite or street-level imagery.

## 2 Related work and contributions

Advances in deep learning methods and increasing availability of satellite and street level imagery led to an increasing number of studies that focus on various applications of deep learning for measurements of the environment. In our review, we focus on studies that have applied deep learning methods to satellite or street level images; subcategories are based on the type of input imagery data. We excluded studies that have used methods other than deep learning and other sources of big data (e.g. mobile phones, activity trackers).

### 2.1 Applications of satellite imagery

Land use classification is one of the most popular applications of deep learning (CNNs in particular) to satellite imagery (Castelluccio et al., 2015; Penatti et al., 2015; Romero et al., 2016; Papadomanolaki et al., 2016; Liu et al., 2018; Yang and Newsam, 2010; Uba, 2016; Albert et al., 2017); some with a specific focus on detection of roads and buildings more specifically (Mnih, 2013; Mnih and Hinton, 2010; Yue et al., 2015; Marmanis et al., 2018; Yuan, 2016). Satellite data is also used for making socio-economic and environmental measurements: poverty detection (Jean et al., 2016; Steele et al., 2017; Xie et al., 2015), measuring health, wealth, population density, and other census based indicators (Engstrom et al., 2011; Sandborn and Engstrom, 2016; Bonafilia et al., 2019; Chew et al., 2018), detecting slum areas (Engstrom et al., 2015), air pollution (Zhang et al., 2018; Chakma et al., 2017; Hong et al., 2019), and infrastructure quality assessments (Oshri et al., 2018). Most are focused on the use of a single source of data, i.e. satellite images from a single source for the target measurement task.

### 2.2 Applications of street-level imagery

Street-level images were successfully used with CNNs for land use classification (Zhu and Newsam, 2015), semantic segmentation (Martinovic et al., 2015), income and voting patterns (Gebru et al., 2017), crime and perceptions of safety (Naik et al., 2014; Arietta et al., 2014), urban density and housing prices (Arietta et al., 2014; Law et al., 2019; Glaeser et al., 2018), urban change (Naik et al., 2017), neighbourhood walkability (Yin and Wang, 2016) and social and environmental inequalities (Suel et al., 2019).

### 2.3 Joint use of satellite and street-level images

One line of work focused on the problem of geolocalization of street level imagery by making use of aerial images (Workman et al., 2015), and geolocalization of aerial imagery using OpenStreetMaps data (Costea and Leordeanu, 2016). These applications focus on enrichment of existing imagery with spatial information via the use of complementary datasets. Prediction of ground level scene images from corresponding aerial images was also investigated (Zhai et al., 2017). Recently, Barbierato et al. (2020) compared greenery metrics and found that those derived separately from street-level images are complementary to ones derived from remote sensing. Cao et al. (2018) used a framework for land use classification with applications to Brooklyn and Queens in New York. Features extracted (using pre-trained networks) from street-level images were used as additional information are subsequently merged with aerial images. These methods, however, are not capable of joint training i.e. networks are separately trained for street-level and remote sensing or aerial images. In land use classification applications, pixel level class labels are available. In our applications, the outputs (income, overcrowding, environmental deprivation) are available as decile classes where there is an ordinal relationship between each class and labels are only available at area-level.

In this paper, we focus on two visual measurement techniques that combine information from satellite and street level images to detect variation in selected output measures at city scale. The first approach builds on two very recent studies by Costea and Leordeanu (2016) and Srivastava et al. (2019) where aerial images are used with street images for dengue incidence rates and land use classification respectively. In this approach, raster tiles cropped from satellite images are appended to street-level images within a deep learning framework. The predictions, however, are limited to locations where street level images are available.

We propose a second technique, novel to the best of our knowledge, capable of making pixel level predictions for all high resolution grid cells in a city (e.g 3m×3m in our experiments), hence not limited to locations where street-level images are available. Our proposed method is capable of jointly training on information coming from two different sources of imagery. It enhances overall prediction performance of satellite imagery at all pixels by utilizing information from available street-level images. We demonstrate its use on measures of income, overcrowding, and living environment deprivation in London. We show that measurement performances improve when bimodal models are used. The proposed methods could be easily extended to incorporate additional modalities of data (e.g. varying numbers of street level images, satellite data from different sources at varying resolutions, aerial images, sound information, mobility data).

Our three main contributions are as follows. First, our proposed approach is the first deep learning-based measurement model that combines information jointly from satellite and street level images to make grid cell (pixel-level) predictions at high resolution and spatial coverage. Specifically, it is capable of joint training on images coming from street-level cars and satellites. Second, our study is the first to show satellite and street-level images have complementary information value that leads to improved performance both for pixel-level predictions covering the entire city and sampled point-level predictions for locations where street imagery is available. Third, our study is the first to combine satellite and street level imagery with the goal of measuring urban inequalities in income, overcrowding, and environmental deprivation.

## 3 Methods: Combining street level and satellite imagery

### 3.1 Problem formulation

Our goal is to make measurements for London using visual information captured by street level and satellite images. Following from our previous work (Suel et al., 2019), images and their geolocations are inputs and outputs are ten decile classes of census variables provided at lower super output area level (LSOA; average population of 1614 with a total of 4833 LSOAs in London). Deciles were computed separately for each outcome, i.e. income, overcrowding, living environment deprivation. The first decile corresponds to worst-off and tenth to best-off areas in the city. Previous work showed that information from satellites and street level images both separately contain information on income, population density, and environmental attributes (Jean et al., 2016; Steele et al., 2017; Xie et al., 2015; Sandborn and Engstrom, 2016; Arietta et al., 2014; Gebru et al., 2017; Suel et al., 2019). Our method can be applied to other outcome measures and regression where the output (label) data are continuous.

Raster tiles from satellite images at one location can be seen as an additional image from a bird's eye view to existing street-level images. Similarly, street-level images taken at one location are additional input to one pixel in a satellite raster essentially appending a layer of visual information. Building on these principles, we propose two multimodal deep learning approaches: SATinSL (augmented street-level network) and SLinSAT (augmented satellite network). The details for each are provided in the following subsections. We compare prediction accuracy of proposed multimodal (i.e. using both street-level and satellite images) and corresponding unimodal (i.e. using only street-level (SL) or satellite images (SAT)) learning approaches.

### 3.2 Augmented street-level network: SATinSL

**Fig. 1:**
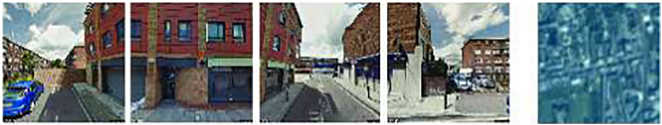
Four street-level image cut outs and corresponding satellite raster tile for one location. Images courtesy of Google and Planet.

In our first method, satellite raster tiles corresponding to street-level image locations are seen as capturing additional visual information from the bird's eye view. For each location, four street-level image cut outs are available corresponding to a camera direction of 0°, 90°, 180°, 270° relative to the vehicle to cover a 360° view. The satellite raster tile for a given location is the fifth input image to the neural network (Fig. 1).

#### 3.2.1 Unimodal model: SL

**Fig. 2:**
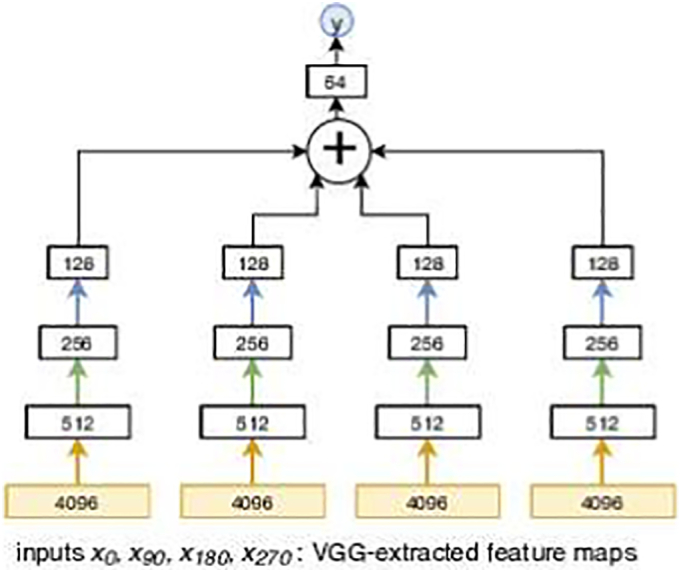
The architecture of the street-level network *M*_*SL*_. Connections with the same colour represent shared weights.

The unimodal approach estimates a function *M*_*SL*_ that makes a prediction of output decile *y* given street-level images only (Eq. (1)). Four street-level images, each with three channels (red-green-blue), from the same location are used as input to the network presented in Fig. 2.

(1) $$y=M_{\mathrm{SL}}\left(x_{0},x_{90},x_{180},x_{270}\right)$$

Following from work by Suel et al. (2019), we use a transfer learning approach for the first part of the street-level only network. We used the first layers of a VGG16 network until *fc*6 (Simonyan and Zisserman, 2014) trained with ImageNet (Russakovsky et al., 2015), and extended it with a smaller fully connected neural network trained from scratch for our prediction tasks. Empirically, training from scratch the entire network did not lead to performance gains; we kept the architecture with transfer learning presented in Fig. 2. The output from the pre-trained VGG16 network for each image location consisted of four 4096 dimensional vectors, which are used as inputs to the fully connected layers. The main principle behind the architecture of the fully connected network is to use shared weights for the codes of images acquired from different camera directions at the same location, considering no angle is preferred over the other. The first four layers summarize the information separately and the smaller 128 dimensional summaries are aggregated by averaging. Size of the layers and the overall architecture was determined empirically using validation data.

#### 3.2.2 Multimodal model: SATinSL

**Fig. 3:**
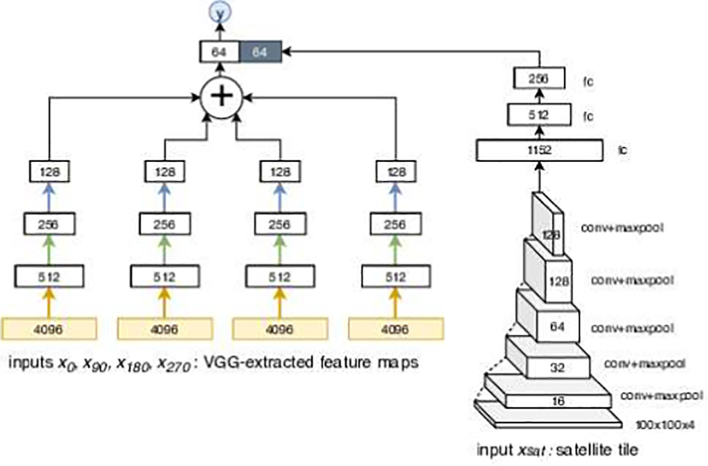
The architecture of the augmented street-level network *M*_*SATinSL*_.

The multimodal model that integrates information from satellite images for prediction at street level consisted of estimating a second function *M*_*SATinSL*_, where the satellite raster tile, *x*_*sat*_ is used as a fifth input with four channels to the network in addition to street-level images (Eq. (2)) as shown in Fig. 3.

(2) $$y=M_{\mathrm{SATinSL}}\left(x_{0},x_{90},x_{180},x_{270},x_{\mathrm{sat}}\right)$$

In the multimodal architecture, we kept the street-level network as is from *M*_*SL*_ so that the resulting unimodal and multimodal performances can be compared. The architecture for convolutional layers for the satellite raster tile was inspired by the VGG architecture. Five convolutional blocks, each consisting of two convolutional layers with 3×3 kernels and zero padding to keep the channel size fixed followed by rectified linear units and a final max-pooling layer, reduce the size of the input satellite tile from 100×100 with four channels to 128 channels each of size 3×3. This tensor is flattened and processed with three fully connected layers yielding a 64 dimensional vector that represents the information in the satellite tile. The 64 dimensional vector from the satellite tile is concatenated to the 64 dimensional vector from street-level images, which were then fed into final layers for the final decile prediction. Details of the satellite part of the architecture were determined empirically using cross-validation on the training set.

The cost function for training both networks is formulated as an ordinal classification task as there is a natural ordering of the outcome classes in our application. We used the ordinal classification approach proposed by Da Costa and Cardoso (2005), which defines a set of Bernoulli trials, one less than the number of total deciles, based on the single continuous variable *v*. Making the analogy with a coin toss, the probability of getting *t* heads is defined as the probability of the *t* + 1^*th*^ = *m*^*th*^ decile, where *t* takes values between 0 and 9 and *m* are the deciles taking values between 1 and 10. The process can be extended to larger number of ordinal classes by changing the number of trials. To train the unimodal as well as the multimodal models, we use cross entropy loss function Eq. (3) where *c*_*n*_ is a one-hot encoded vector label for the *n*^*th*^ sample, *c*_*n*_^*m*^ its *m*^*th*^ component, and *P*_*n*_^*m*^(*v*) is the probability of the *m*^*th*^ decile for the *n*^*th*^ computed from the Bernoulli trials.

(3) $${ℒ}_{\mathrm{SL}}=\sum_{n}^{N}\sum_{m=1}^{10}c_{n}^{m}\ln P_{n}^{m}\left(v\right)$$

### 3.3 Augmented satellite network: SLinSAT

**Fig. 4:**
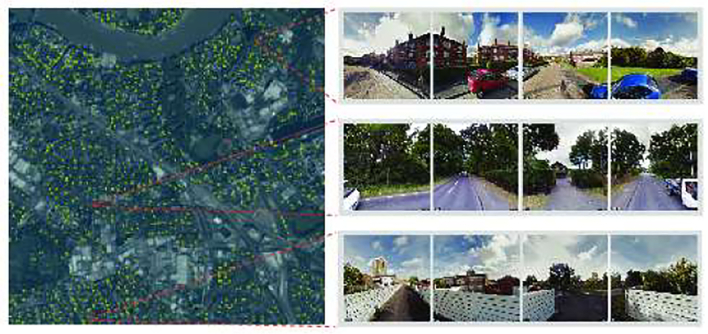
Satellite raster tile example from London. Street-level images are available for locations highlighted with yellow pixels. For three locations, the corresponding street-level images are also shown. Images courtesy of Google and Planet. (For interpretation of the references to colour in this figure legend, the reader is referred to the web version of this article.)

As our proposed second approach, we consider street-level images at one location a providing additional information to that of the corresponding pixel in the satellite image (Fig. 4). Only very few pixels in a satellite image have a corresponding street-level image, but our proposed method is capable of accommodating such sparsely available data. The street-level images essentially form an additional layer (channel) of visual information in addition to what is already captured by the satellites. In contrast to the first approach presented in Section 3.2, outcome predictions *y* are available for each pixel in a satellite image (e.g. for each 3*m*^2^ grid cell for London in our experiments).

#### 3.3.1 Unimodal model: SAT

The unimodal satellite network *M*_*SAT*_ is a pixel-wise prediction function. The input is a satellite tile *x*_*sat*_ of any size *w* x *h*, and the output is a decile raster *r*_*y*_ of the same size *w* x *h* where each pixel contains a single decile value *y* that can take a value between 1 and 10.

(4) $$r_{y}=M_{\mathrm{SAT}}\left(x_{\mathrm{sat}}\right)$$

**Fig. 5:**
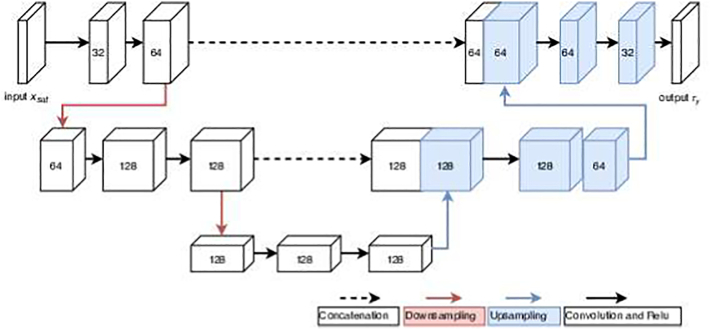
The 2D U-Net architecture used for the unimodal satellite network *M*_*SAT*_. The input *x*_*sat*_ has four channels for the satellite data. Red arrows indicate downsampling and blue upsampling operations. (For interpretation of the references to colour in this figure legend, the reader is referred to the web version of this article.)

We model *M*_*SAT*_ with a 2D U-Net architecture (Ronneberger et al., 2015) as shown in Fig. 5. We used 3×3 convolutions with zero padding to retain the image size throughout the network, rectified linear units as activation functions, pooling layers with stride 2 for down-sampling and bi-linear up-sampling followed by convolution to increase the resolution. The details of the architecture was determined empirically on the validation set.

#### 3.3.2 Multimodal model: SLinSAT

**Fig. 6:**
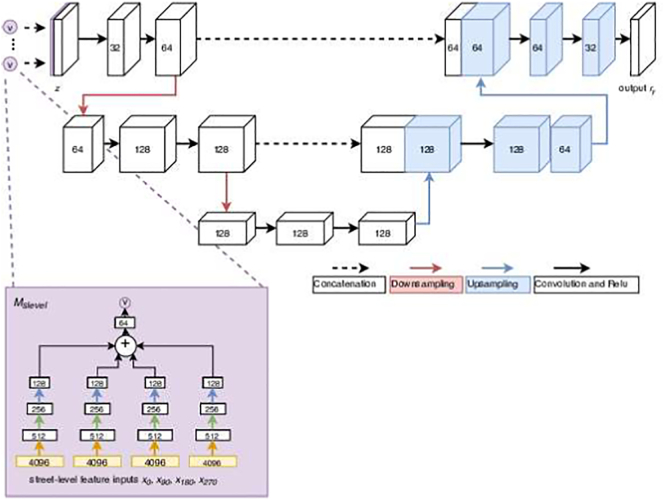
The architecture used for the multimodal satellite network *M*_*SLinSAT*_.

The augmented multimodal satellite network, *M*_*SLinSAT*_ is also a pixel-wise prediction function as shown in Fig. 6. The inputs are a satellite tile of any size *w* x *h*, and street-level images for the pixels where this information is available. The number of pixels with street-level images will depend on their availability for the spatial extent of the satellite tile. For each of the available pixels, four cut outs are used corresponding to four camera directions as described in the previous section.

The first part of the network aims to summarize street-level information and feed it to the U-Net architecture in the second part. The first part estimates *M*_*SL*_ (Eq. (5)) using the same architecture as *M*_*SL*_ presented in Section 3.2 but the output is different. *M*_*SL*_ takes in four street-level images and summarizes this information to a one-dimensional feature vector *v*. The outputs *v* from *M*_*SL*_ are appended as an additional channel to the original satellite image. For pixels where no street-images are available, the channel value is missing. This results in a street-level layer that have many missing values and others having the value *v* derived from the corresponding street level image from that location. The same network is used for all the pixels with street level images, weights of the network that processes street level images are shared across the pixels of the satellite image.

(5) $$v=M_{\mathrm{SL}}\left(x_{0},x_{90},x_{180},x_{270}\right)$$

The second part of the network uses an identical architecture to that of *M*_*SAT*_, with one difference: the input is a multi-channel image *z* that is obtained by concatenating the channels from the original satellite image *x*_*sat*_ and *v* from Eq. (5). This part estimates the function *M*_*SLinSAT*_ that uses the combined information and creates a decile raster *r*_*y*_ (Eq. (6)).

(6) $$r_{y}=M_{\mathrm{SAT}}\left(z\right)=M_{\mathrm{SAT}}\left(x_{\mathrm{sat}},v\right)=M_{\mathrm{SAT}}\left(x_{\mathrm{sat}},x_{0},x_{90},x_{180},x_{270}\right)$$

The number of channels in *z* will be determined by number of channels available from the satellite image *x*_*sat*_ and the size of the vector output *v*. In our application and experiments, the size of *z* was five, four coming from the satellite image and one from *v*. The formulation also easily extends to a case where more bands are used from available satellite data and extracted from street-level images. Both parts of the network (i.e. both *M*_*SL*_ and *M*_*SLinSAT*_) are trained jointly as explained in more detail in Section 5.

Similar to the augmented street-level network, the augmented satellite network was also trained using the ordinal classification loss, but at the pixel-level this time with a sum over all pixels *p* in the image domain Ω (Eq. (7)).

(7) $${ℒ}_{\mathrm{augsat}}=\sum_{n}^{N}\sum_{p\in \Omega }\sum_{m=1}^{10}c_{n}^{m}\left(p\right)y_{n}^{m}\left(p\right),$$

where *y*_*n*_^*m*^(*p*) = *P*_*n*_^*m*^(*M*_*SLinSAT*_(*z*))|_*p*_ and *c*_*n*_ corresponds to the labels of the satellite data.

### 3.4 Study design

Merging outcome labels, satellite and street-level images required the use of geographic information system (GIS) tools. We converted individual sets of data to raster images that contain spatial information with a pixel resolution of 3 m in line with the satellite images obtained from Planet (Planet Team, 2017). We used the spatial information in raster images for matching to ensure pixels in each raster will correspond to the same geographic location. Temporal differences in data collection were not taken into account in these experiments. For outcome data, we used the most recent data available. For satellite images, it was not possible to get historical data as Planet data at high resolution was only available for the past few years, the images were chosen based on minimum cloud coverage as detailed below.

## 4 Data

We used data from London to evaluate the feasibility of our proposed method for combining street-level and satellite imagery. For the experiments presented here our task was making measurements at the satellite image's resolution and extent relating to inequalities in three chosen domains: income, overcrowding, and living environment deprivation. Collection and processing of the data sets are explained in detail in the following subsections.

### 4.1 Annotated maps as labels

Outcome data was obtained from the UK Census 2011 (ONS, 2011) for overcrowded households, Greater London Authority (Greater London Authority (GLA), 2015) for income, and English Indices of Deprivation for living environment deprivation (Ministry of Housing, Communities, and Local Government, 2015). The lowest level of geography where all outcome data were available was LSOA. For income, we used the mean annual household income estimates. For overcrowding, we used the percentage of households classified as being overcrowded as determined by the Office for National Statistics (ONS) and defined as having at least one fewer room than required based on the number of occupants. For living environment deprivation, the corresponding index was used capturing air quality, traffic crash rates, and housing in poor condition. For all three outcomes, we calculated deciles of LSOAs in London, with decile 1 corresponding to the worst-off 10% and decile 10 to the best-off 10%. We generated separate LSOA maps for each outcome as raster images, where each pixel contained a decile value.

### 4.2 Street-level images

Street level images were sourced from Google Maps using the Google Street View API. For each postcode in London (ONS, 2017), the API returned the unique identifier for the nearest available panorama image most recently taken by Google, if available. The time stamp ranged from 2008 to 2018. Panorama images were available for 145,756 of the postcodes corresponding to 119,238 unique panoids. We used four image cut outs for each panorama by specifying the camera direction (i.e. 0°, 90°, 180°, 270°) relative to the vehicle to cover a 360° view. We extracted 4096 dimensional codes from each of the four images using pre-trained VGG16 (Simonyan and Zisserman, 2014; Russakovsky et al., 2015) network weights as explained in Section 3.1.

### 4.3 Satellite images

Satellite imagery was obtained from Planet (formerly Planet Labs) free of charge, through their education and research program (Planet Team, 2017). Planet is one of the commercial companies that manages and have launched small light weight satellites i.e. CubeSats. The images freely available from Planet are taken by CubeSats that are capable of capturing RGB and near infrared (NIR) imagery at three meter ground sampling distance. We obtained 44 cloud free images taken on 27 September 2018 between 9 am to 10 am and cover the full area of Greater London Authority. We created a single mosaic dataset of size 19672x15299x4 for London including RGB and NIR bands, with a pixel resolution of 3 m. Range of the image intensity from satellites images were high and were stored as 16 bit unsigned integer. The labels were only available for the administrative boundaries of Greater London, so not all pixels were labeled. The cost function does not take these unlabeled pixels into account when computing the loss.

## 5 Experiments

### 5.1 Evaluation

We evaluated the proposed method using the data collected from London, detailed in Section 4. Specifically, we wanted to test the hypothesis that a multimodal framework combining visual information from both satellite and street-level images will help improve prediction performance compared to unimodal frameworks that exist in the literature (Andersson et al., 2019; Srivastava et al., 2019). For this purpose, we compare the performances of street-level only *M*_*SL*_ and satellite only *M*_*SAT*_ networks with the proposed two alternative approaches: *M*_*SATinSL*_ and *M*_*SLinSAT*_.

First is a comparison between *M*_*SL*_ and *M*_*SATinSL*_. We start with a unimodal network for street-level images only and use architecture *M*_*SL*_. For *M*_*SATinSL*_, the size of the satellite image can take any value; we use 100×100 pixel tiles for our experiments. This tile captures a 300m×300m area around the coordinate location for the street-level images from a bird's eye view.

Second is a comparison between *M*_*SAT*_ an *M*_*SLinSAT*_. The unimodal network *M*_*SAT*_, for satellite images only makes pixel-level predictions for all pixels covering Greater London, each corresponding to a 3m×3m area. For *M*_*SLinSAT*_, information from street-level images are also included. Not all pixels had the additional information from a street-level image due to their comparatively sparser availability.

To generate test and training splits, we took the following steps. We partitioned the data by generating 320 non-overlapping tiles of size 1000×1000 pixels (corresponding to a 3 km × 3 km ground area) from the initial Greater London satellite mosaic from Section 4.3. We did this because holding out large chunks of non-overlapping areas makes prediction harder compared to random hold-out. Each of these tiles were matched with street-level images using coordinates. For evaluation, we used four-fold cross validation. In each fold, 75% of data (i.e., image-outcome pairs for 75% of 320 non-overlapping tiles) were used for training the network and the remaining 25% were withheld. We then measured how well the trained network uses images to predict outcomes at locations that were not used in training. We repeated this process four times holding out a different 25% of data each time. We used stratified sampling when generating splits to ensure equal representation of street-level images from each of the decile classes in training and testing sets. Section 5.3 report the average test performances, as well as ranges and standard deviation of performances across cross-validation runs.

### 5.2 Training

For *M*_*SL*_, we trained fully connected layers shown in Fig. 2. For *M*_*SATinSL*_ we jointly trained the convolutional and fully connected layers for the satellite part of the network and the fully connected layers for the street-level part of the network shown in Fig. 3. For both, we used the loss function given in Eq. (3) for training, and validation sets for monitoring the training. Both for street level and satellite images, we normalized intensities to have mean 0 and standard deviation 1, using means and standard deviations computed over training sets. Satellite image intensities were log-transformed before normalization. Satellite tiles, *x*_*sat*_, tile consisted for four channels: RBG and NIR channels from Planet data. For training *M*_*SAT*_ and *M*_*SLinSAT*_, we extracted 200 × 200 pixel tiles randomly from the 1000×1000 pixel tiles at each iteration during training.

*M*_*SL*_ and *M*_*SATinSL*_ models were trained for 20 epochs while *M*_*SAT*_ and *M*_*SLinSAT*_ were trained for 500 epochs. At the end of each epoch, we computed the error on the validation set and saved the model with the minimum validation error. We used Adam (Kingma and Ba, 2014) with a learning rate of 0.0001 for all the experiments. To avoid overfitting we used a weight decay of 0.0001 in all the experiments. We used PyTorch in our implementation. The dynamic computation graph construction was essential for building *M*_*SLinSAT*_ models. The same model would not have been possible when using a static computation graph construction.

### 5.3 Results

For quantitative evaluations, ground truth data was only available at the LSOA level. Therefore, for computing test errors for evaluations, we computed LSOA level predictions as the average of location level predictions assigned to that LSOA. We computed mean absolute errors (in units of decile classes) separately for each of the four cross validation runs. We report the averages of the four cross validation runs along with minimum and maximum values for each outcome.

**Fig. 7:**
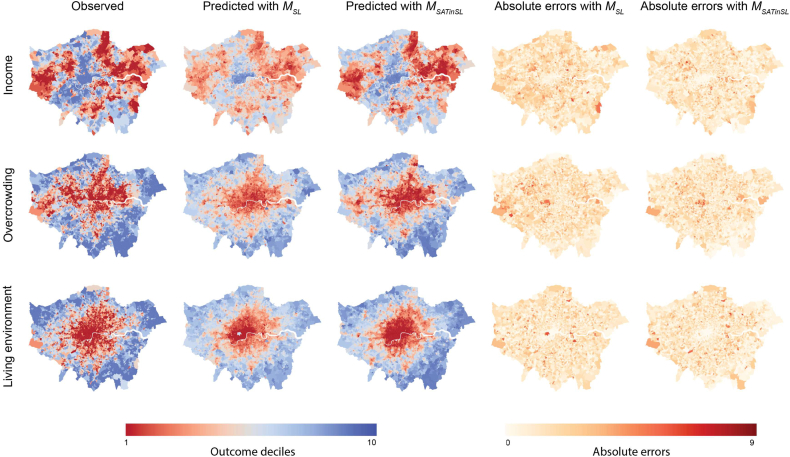
Comparison of ground truth and prediction maps generated by *M*_*SL*_ and *M*_*SATinSL*_. *M*_*SL*_ used street-level images only for generating these maps. *M*_*SATinSL*_ made use of both satellite and street-level imagery, and enhanced measurement performances as observed by predicted decile maps. Maps are colour coded where red correspond to worst-off deciles and blue correspond to best-off deciles. Ground truth (observed) decile maps are also presented for comparisons. Absolute error maps are also presented. (For interpretation of the references to colour in this figure legend, the reader is referred to the web version of this article.)

For comparing performances of *M*_*SL*_ and *M*_*SATinSL*_, we computed LSOA level predictions as the average of predictions made at locations where street-level imagery was available. The mean absolute error between true and predicted LSOA classes for both approaches are shown in Table 1. The multimodal approach outperformed the unimodal one for all outcomes. The error rates for all three outcome predictions were reduced when using the additional information coming from satellite tiles. Use of satellite data led to highest performance gains for mean income, yet interpretation of such differences is outside the scope of this paper and will require a dedicated study. Fig. 7 show observation and prediction maps from *M*_*SL*_ and *M*_*SATinSL*_ approaches for the whole study area i.e. Greater London, separately for each outcome measure. These maps show that *M*_*SATinSL*_ can better capture the spatial patterns for each of the outcomes. For example, *M*_*SL*_ prediction map clearly shows that Hyde Park is predicted as having high quality living environment i.e. blue (e.g. low air pollution) when using street-level images only. This is in line with intuition, as the network does not have any information on the relative location of the park when only observed from a street point of view (e.g. capturing trees from inside the park). *M*_*SLinSAT*_ does a better job with this area, as the satellite tile helps the network to recognize Hyde Park is,in fact, located within the city center hence will have poorer air quality relative to a park located elsewhere in the urban area. A similar pattern is also observed for the area around the Heathrow Airport.

**Table 1:** Mean absolute error (MAE) of four-fold cross-validation experiments, comparing unimodal (street-level only) and multi-modal (street-level images augmented by satellite tiles) approaches.

Method	Income	Overcrowding	Living Environment
	Mean [Min-Max]	Mean [Min-Max]	Mean [Min-Max]
*M*_*SL*_	1.54 [1.51–1.58]	1.45 [1.40–1.53]	1.28 [1.25–1.36]
*M*_*SATinSL*_	1.23 [1.18–1.30]	1.30 [1.19–1.41]	1.17 [1.14–1.22]

For comparing performances of *M*_*SAT*_ and *M*_*SLinSAT*_, we computed LSOA level predictions as the average of predictions made at each pixel from *r*_*y*_ within that LSOA. Table 2 shows the mean absolute error using true and predicted LSOA classes for both approaches. The multimodal approach outperformed the unimodal one for all outcomes. The error rates for all three outcome predictions were reduced when using the additional information coming from street-level imagery.

**Table 2:** Mean absolute error (MAE) of four-fold cross-validation experiments, comparing unimodal (satellite only) and multi-modal (satellite tiles augmented by street-level images) approaches.

Method	Income	Overcrowding	Living Environment
*M*_*SAT*_	1.73 [1.61–1.84]	1.62 [1.49–1.64]	1.63 [1.59–1.67]
*M*_*SLinSAT*_	1.63 [1.57–1.70]	1.45 [1.36–1.56]	1.45 [1.32–1.53]

**Fig. 8:**
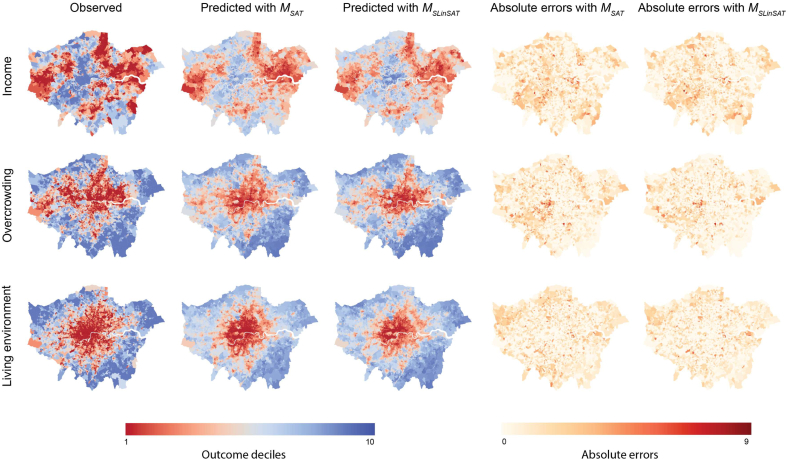
Comparison of ground truth and prediction maps generated by *M*_*SAT*_ and *M*_*SLinSAT*_. *M*_*SAT*_ used satellite images only for generating these maps. *M*_*SLinSAT*_ made use of both satellite and street-level imagery using our proposed approach, and enhanced measurement performances as observed by predicted decile maps. Maps are colour coded where red correspond to worst-off deciles and blue correspond to best-off deciles. Ground truth (observed) decile maps are also presented for comparisons. Absolute error maps are also presented. (For interpretation of the references to colour in this figure legend, the reader is referred to the web version of this article.)

Fig. 8 show observation and prediction maps from *M*_*SAT*_ and *M*_*SLinSAT*_ approaches for the whole study area i.e. Greater London, separately for each outcome measure. As expected, on average for LSOA level performances, both approaches presented in Table 2 do worse when compared to Fig. 7 and Table 1 as the predictions are made for each of the pixels, and there is more observations and variability within a given LSOA. The improvement from *M*_*SAT*_ to *M*_*SLinSAT*_ are spatially visible from Fig. 8 and from Table 2.

**Fig. 9:**
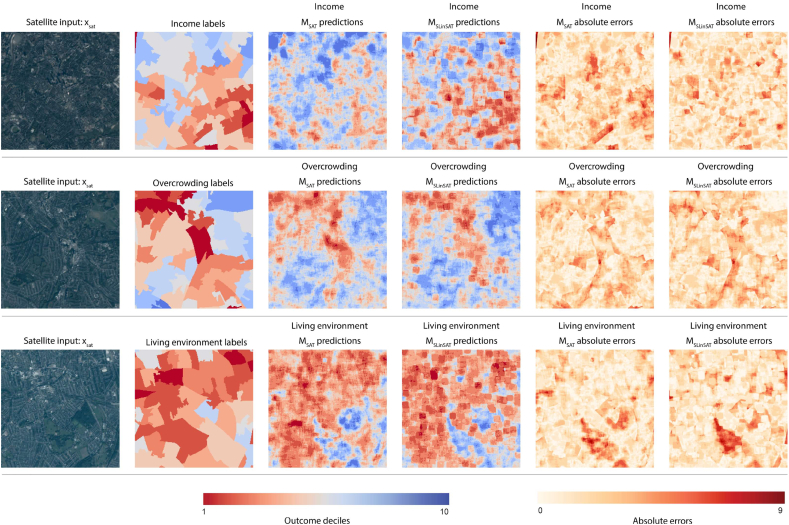
Comparison of ground truth and prediction maps generated by *M*_*SAT*_ and *M*_*SLinSAT*_. *M*_*SAT*_ used satellite images only for generating these maps. *M*_*SLinSAT*_ made use of both satellite and street-level imagery using our proposed approach for combining them, and enhanced measurement performances as observed by predicted decile maps. Maps are colour coded where red correspond to worst-off deciles and blue correspond to best-off deciles. Ground truth decile maps (labels) are also presented for comparisons along with the original satellite images. Street level images are used for predictions that were available for the geographic area covered by each tile. Individual street level images are not displayed in the figure due to space limitations. Images courtesy of Planet. (For interpretation of the references to colour in this figure legend, the reader is referred to the web version of this article.)

Examples of the estimated income and overcrowding maps from test tiles are shown in Fig. 9 generated both from *M*_*SAT*_ and *M*_*SLinSAT*_. The visual figures demonstrate how additional information from street-level images combined with satellite data can improve output maps. Prediction surfaces from *M*_*SLinSAT*_ show that there are block effects. We can only utilize street-level information at locations where images are available resulting in these block effects. Locations with more street-images have smoother prediction surfaces. Spatial interpolation techniques (e.g. Gaussian processes (Suel et al., 2018)) can be explored in future work.

## 6 Discussion and limitations

We proposed a novel deep learning based multimodal framework to jointly utilize satellite and street-level images. Our method enables city wide measurements at high spatial resolution and can utilize city wide availability of satellite images as well as locally rich information coming from street-level images where available. The resolution of predictions are limited only by the resolution of input satellite imagery, and not by availability of street-level images. Building on previous studies, we also use a second multimodal approach for comparison purposes; its training and prediction capability is limited to locations where street-level images are available and cannot fully utilize the city wide availability of satellite imagery.

To the best of our knowledge, this is the first study developing a technique for combining satellite and street-level images to grid level city-wide measurements at high resolution. For our experiments, we use Planet data at 3*m*^2^ resolution hence resulting predictions are available for each 3*m*^2^ grid cell in London. Both methods successfully utilized different information from multimodal imagery data i.e. street-level and satellite images, and outperform measurement performances obtained from unimodal alternatives in our experiments.

Both methods can be extended to make measurements for other outcome variables (e.g. land use classification, green space) and incorporate other sources of images (e.g. aerial images, satellite data at different resolutions with variable bands). Our proposed methods can incorporate additional layers of imagery data such as aerial images or satellite images with different resolutions or additional spectral data. Commercial satellite imagery at higher pixel resolutions of up to 0.3 m are also becoming available for researchers through sponsored challenges (Demir et al., 2018; Van Etten et al., 2018), yet remains very costly even for research purposes.

One limitation of our study is that street and satellite images are available only at specific times, which may be different from when ground truth data used for model training and testing were collected. In our experiments, images and outcome data were a few years apart (see Data for details). That said, model performances were high indicating that images contained visual cues of outcomes measured. Increasing availability of temporally aligned dataset may allow for interpolating in time. Another related limitation relates to utilizing repeated observations from the same location over time. Street-level images from the same locations are often available every 2–4 years for cities. Satellite images are taken repeatedly for several days in a month. It will be ideal to utilize repeated observations coming from the same locations within a single framework. Additional work is also needed to develop multimodal learning methods that can also incorporate other types of big data such as mobile phone tracking, and social medial.

More general limitations exist that relate to the use image data for making urban measurements. Street level imagery is often not available for night time, spatially sparse, and cannot capture indoor environments except for purpose-collected time-lapse images (Clark et al., 2020). Remote sensing data might be hard to capture during the night or cloudy days. While satellite imagery have continuous coverage, the resolution and available bands may provide limited information on certain attributes of the environment. For instance, building facades that can contain valuable information are not captured. Performances are heavily dependent on what is being measured, and to what extent visual cues contain information on the selected outcome measure. Observations (labels) available often do not have high spatial resolution, hence it is not possible to evaluate pixel-level performances. Future work will also investigate how well images perform for increasing the spatial resolution of available data from traditional sources. In this study, we applied our proposed approach to one city only for three selected outcome variables; performances might be higher or lower for different locations and measures.

## Author contributions

ES designed study concept with input from SB, MB, SF, and ME. ES obtained data, conducted analysis, and prepared results with input from SB, MB, SF, and ME. ES, MB, SB, and SF wrote the first draft of the paper. ME contributed to the final paper.

## Data and code availability

All datasets used in this paper are publicly available and the URLs are provided in the Data section. Upon publication, the code will be available at https://github.com/esrasuel/sview-sat-combined and http://equitablehealthycities.org/data-download/

## Declaration of Competing Interest

The authors declare that they have no known competing financial interests or personal relationships that could have appeared to influence the work reported in this paper.
